# Role of prebiotic dietary fiber in periodontal disease: A systematic review of animal studies

**DOI:** 10.3389/fnut.2023.1130153

**Published:** 2023-03-14

**Authors:** Rohan Tailor, Nidhi Medara, Aditi Chopra, Hasinthi Swarnamali, Joerg Eberhard, Thilini N. Jayasinghe

**Affiliations:** ^1^The Charles Perkins Centre, The University of Sydney, Darlington, NSW, Australia; ^2^Sydney Dental School, Faculty of Medicine and Health, The University of Sydney, Darlington, NSW, Australia; ^3^Department of Periodontology, Manipal College of Dental Sciences, Manipal, Manipal Academy of Higher Education, Manipal, Karnataka, India; ^4^Health and Wellness Unit, Faculty of Medicine, University of Colombo, Colombo, Western Province, Sri Lanka

**Keywords:** periodontitis, dietary fiber, systemic inflammation, animal models, cytokines, alveolar bone loss

## Abstract

**Background:**

Periodontitis is a chronic inflammatory condition affecting the supporting structures of a tooth in the oral cavity. The relationship between dietary fiber and periodontitis is poorly understood. The objective of this systematic review is to investigate if an intake of dietary fiber modulates periodontal disease in animal models and any concomitant effects on systemic inflammation, microbiota and their metabolites.

**Methods:**

Animal studies using periodontitis models with any form of fiber intervention were included. Studies with comorbidities that were mutually inclusive with periodontitis and animals with physiological conditions were excluded. Search strategy with MeSH and free-text search terms were finalized and performed on the 22nd of September 2021.CINAHL Complete, EMBASE, MEDLINE, SciVerse Scopus® and Web of Science Core Collection databases were used to identify studies. SYRCLE’s risk of bias tool and CAMARADES were used for quality assessment. Results were synthesized utilizing Covidence© web-based platform software to remove duplicates, and the remaining studies were manually filtered.

**Results:**

A total of 7,141 articles were retrieved from all databases. Out of 24 full-text articles assessed for eligibility, four studies (*n* = 4) were included. Four studies involved the use of *β*-(1,3/1,6)-glucan (*n* = 3) and mannan oligosaccharide (*n* = 1) at differing dosages for different study durations. All studies utilized a ligature-induced model of periodontitis in rats, either Wistar (*n* = 3) or Sprague–Dawley (*n* = 1). A dose-dependent relationship between the increased fiber intake and decrease in alveolar bone loss and pro-inflammatory markers was observed.

**Conclusion:**

The number of included studies is limited and narrow in scope. They highlight the importance of pre-clinical trials in this field with broader dietary fiber intervention groups before proceeding to clinical trials. The use of dietary fiber as an intervention shows promise in the reduction of inflammatory conditions like periodontitis. However, further research is required to delineate the relationship between diet and its effects on microbiota and their metabolites such as short chain fatty acids in animal models of periodontitis.

## Introduction

The ability to masticate, taste and live a life of general well-being is in part dependent on the healthy function of the oral cavity. Periodontitis or gum disease is a prevalent condition affecting 10.8% of the adult population worldwide, accounting for 743 million people (1). Within the Australian adult population in 2018, 3 in every 10 adults aged 15 and above present with moderate–severe periodontitis, a 38% increase from the National Study of Adult Oral Health conducted between 2004 and 2006 (2, 3).

Periodontitis is a chronic immune-inflammatory disease affecting the supporting structures of a tooth, which includes the gingiva, cementum, periodontal ligament, and alveolar bone. The loss of these tissues as a result of the inflammatory response is irreversible, and if left untreated, may ultimately lead to tooth loss and loss of function in the oral cavity. The polymicrobial plaque biofilm adjacent to a tooth is the primary etiological agent for periodontitis. However, disease progression or resolution is a complex interplay between the host immune response, genetic, epigenetic, environmental, and behavioral factors (4). In health, periodontal tissues are in a state of homeostasis where host inflammation and the biofilm prevent destructive inflammatory disease. A shift in the biofilm from a eubiotic to a dysbiotic state leads to altered host-microbial interaction and destructive inflammation. The mechanisms that drive the shift toward dysbiosis are yet to be clarified. However, keystone pathogens such as *Porphyromonas gingivalis* or other putative periodontal pathogens may play a role (5, 6). Clinically, a reduction in biofilm load leads to reduced inflammation, or a reduction of inflammation modifies dysbiosis and prevents tissue destruction (7). The local dysbiosis and inflammation is not only constrained to the oral cavity but may have an effect of other systemic sites mediated by the leakage through the highly vascularised and ulcerated oral epithelial barrier in exacerbated periodontal lesions (8).

During this dysbiosis, there is an increased inflammatory response with upregulated inflammatory mediators such as IL-1, IL-1β, IL-6, IL-18, PGE2, IL-21, TNF-α, MMP-8 and downregulated TIMP-1 (9–15). An upregulation of miRNAs, such as miRNA-7, miRNA-100 and miRNA-125 are associated with early stages of periodontal disease. Moreover, within systemic diseases such as cardiovascular disease, miRNA-3198 and miRNA-4299 are expressed highly within the subgingival plaque of individuals suffering from periodontitis (16). A recent study has further shown the response of other RNA types in an induced model of periodontitis in mice, there is a higher level of mRNA and protein expression of Claudin-1 and Claudin-2 (17).

This highlights the importance of new management methods that need to be implemented as an adjunct to the current gold-standard of scaling and root debridement or professional dental cleaning and patient oral hygiene instructions. Many studies have observed that a balanced diet with proper nutrition plays an essential role in maintaining periodontal health (18, 19) as well as gut and overall health (20). Dietary fiber is a central component in a healthy diet and the Heart Foundation recommends an intake of 30 grams for men or 25 g for women per day (21). Fermentation of dietary fiber, specifically oligosaccharide, polysaccharide and resistant starch by-products by colonic anaerobic microbes produce short chain fatty acids (SCFAs) such as acetate, butyrate and propionate, which have key roles in regulating host metabolism and immunity (22). SCFAs modulate gene expression by inhibiting histone deacetylase impeding DNA transcription of various pro-inflammatory cytokines (23, 24). Acetic acid has been shown to inhibit the production of pro-inflammatory mediators like interleukin (IL)-6, IL-8, IL-1β and tumor necrosis factor-α (TNF-α) in LPS-activated macrophages (25). In mouse models, butyrate induces anti-inflammatory T-regulatory (T-Reg) cell proliferation outside the thymus and propionate induces peripheral T-Reg cell generation (26). In animal models of other inflammatory diseases such as colitis, acetate from a high fiber diet binding to the G-protein receptors 43 and 109A reduces disease severity whilst diets low/deficient in dietary fiber exacerbates colitis development (27).

To the best of our knowledge, there is no comprehensive review investigating the relationship between dietary fiber intake and periodontal disease focusing on the SCFAs, oral and gut microbiome, and their effects on the systemic inflammation. We hypothesized that with an ingestion of a higher fiber diet there will be a lesser severity of periodontitis *via* the systemic anti-inflammatory effect of SCFAs compared to a diet consisting of a low fiber diet. The aim of this systematic review was to collate the current evidence about the relationship between dietary fiber and periodontitis within animal models to answer two specific questions. (1) What is the effect of dietary fiber on animal models of periodontitis? (2) Does dietary fiber modulate periodontal inflammation *via* the host microbiota and its metabolites? This review has the capacity to identify dietary fibers as a powerful tool for managing periodontal disease and can inform hypothesis-driven pre-clinical and clinical trials.

## Methods

This systematic review was registered with PROSPERO (CRD42021282362) and conducted according to the Preferred Reporting Items for Systematic Reviews and Meta-analysis (PRISMA) guidelines (28).

### Eligibility criteria

#### Inclusion criteria

Studies with periodontitis induced animal models by any method, both female and male were included. Additionally, studies involving dietary fiber, defined as any fiber that cannot be absorbed by the gastrointestinal digestive system, as the intervention group *via* any route of administration were eligible for inclusion. Any studies where dietary fiber was combined with other supplements and compared against groups with dietary fiber alone were also included. All studies where laboratory animals with periodontitis with dietary fiber as the intervention are compared to any placebo or no intervention group (controlled variable) were also included. Study designs included were randomized controlled trials, quasi randomized trials, analytical studies, pre-clinical trials (e.g., cross-over studies, sequential feeding trials, parallel studies, longitudinal trials). All study durations were also included. Studies with at least one of the following primary outcomes were included: alveolar bone loss and radiographic changes, clinical attachment level/loss, bleeding on probing, gingival bleeding index, clinical probing depth/pocket probing depth, gingival index, plaque index, gingival recession depth, periodontal inflamed surface area index. Secondary outcomes including changes in the microbiota, inflammatory biomarkers and metabolites in serum, blood, stool samples, gingival crevicular fluid or oral tissues upon dietary fiber intake were also included.

#### Exclusion criteria

Studies with comorbidities that are mutually inclusive with periodontitis and animals with physiological conditions (e.g., pregnancy) were excluded. Case reports, case series, letters to the editor, reviews, early view articles, studies without a control/comparator group, observational studies, *ex-vivo*, *in-silico,* and studies in cell lines were also excluded. Any study that used fiber in a combined intervention or its impact is unable to be verified by itself were excluded. Studies where dietary fiber in any form are not used as an intervention in any of the groups will be excluded. Studies without at least one primary and/or secondary outcomes mentioned above were excluded.

### Information sources and search strategies

Electronic searches were conducted in a stepwise process in the following databases: CINAHL Complete (EBSCO, United States), EMBASE *via* OvidSP (Elsevier Properties, Netherlands), MEDLINE *via* Ovid (National Library of Medicine, United States), SciVerse Scopus® (Elsevier Properties S.A, United States) and Web of Science Core Collection databases (v.5.4, Thomson Reuters, United States). A comprehensive search strategy for electronic databases was developed (Supplementary Table S1). The search terms included a combination of controlled terms (MeSH terms) and free text terms for both dietary fiber and periodontitis in animal models. Additionally, manual searching on Google Scholar and citation tracking was performed to retrieve relevant articles. Preliminary searches were performed from 1st September to 17th September 2021 and a comprehensive search strategy with MeSH and free text search terms was finalized and performed on the 22nd September 2021. A simple Google Scholar search was performed on the 28th September 2022 to search for any new articles between September 2021 and September 2022. There were no restrictions on the search for language and publication period to ensure that all relevant publications were reviewed. Searches were re-run prior to the final analysis and any further studies identified were retrieved for inclusion. Unpublished data were not included in this systematic review.

### Data extraction

Covidence (29), a web-based software, was used for article screening and data extraction. Title and abstract screening, full text review and data extraction were performed by 2 reviewers (RT and TJ). The following data was extracted: *study design:* country; *animal model*: species, sex, sample size, method of periodontitis induction; *intervention of interest*: details of intervention and control conditions including type of fiber, fiber dosage, route of administration, duration of intervention and frequency of administration. *Primary periodontitis outcomes* included clinical parameters such as alveolar bone loss (ABL), radiographic changes, clinical attachment level or clinical attachment loss (CAL), bleeding on probing, gingival bleeding index, clinical probing depth, gingival index, plaque index, gingival recession, periodontal inflamed surface area index, statistically significant impact of fiber on periodontal disease parameters when compared to the control group’s *p*-values and confidence intervals (continuous), or suggested mechanisms of intervention action. *Secondary periodontitis outcomes* included changes in the microbiota, their metabolites or inflammatory biomarkers in serum, blood, stool, gingival crevicular fluid or oral tissues upon dietary fiber intake, or statistically significant impact of fiber on periodontal disease parameters when compared to the control group’s *p*-values and confidence intervals.

### Quality assessment

The included studies were independently assessed for any study quality risks of biases by two reviewers (RT and TJ) utilizing the Systematic Review Centre for Laboratory animal Experimentation’s (SYRCLE’s) [adapted from (30)] and the Collaborative Approach to Meta-Analysis and Review of Animal Data from Experimental Studies (CAMARADES) [adapted from (31, 32)] checklists.

## Results

### Results of the search

**Figure 1:**
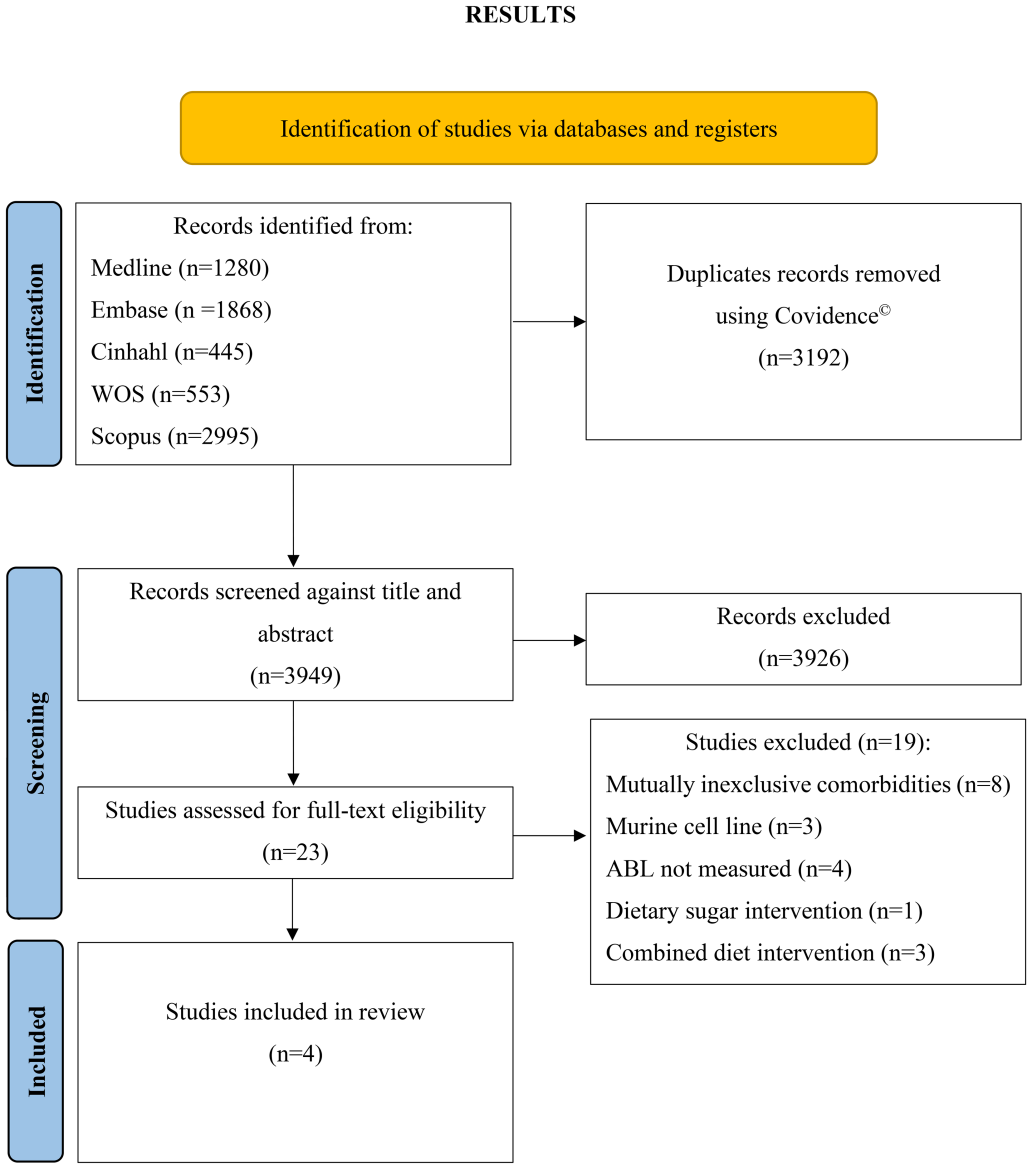
PRISMA 2020 flow diagram for new systematic reviews which included searches of databases and registers only. WOS, Web of Science; ABL, alveolar bone loss.

The initial search across all five databases yielded 7,141 studies when searched for fiber AND periodontitis AND animal* search terms (Figure 1). This initial search was conducted independently by two investigators (RT and TJ) and confirmed upon discussion. Results were collated in EndNote and imported into Covidence where 3,192 duplicates were removed. The remaining 3,949 studies were screened individually by two investigators (RT and TJ) against title and abstract, of which 3,926 were excluded. The remaining 23 studies were screened in entirety by two independent investigators (RT and TJ) and 19 studies excluded. The reasons for exclusion these 19 studies are as follows: 8 studies used mice with mutually non-exclusive or physiological condition in addition to periodontitis [(pregnancy, *n* = 1) or comorbidities (ovariectomized, *n* = 4; diabetes, *n* = 2; inflammatory arthritis, *n* = 1)], 3 studies utilized a murine cell line, 2 studies measured osteoclast differentiation, 1 study measured the efficacy of a hydrogel drug carrier, 1 study measured the proliferation of gingival mesenchymal stem cells, 1 study investigated a dietary sugar supplement in the form of oligosaccharides, 1 study had an intervention with a combined diet, and 2 studies utilized a topical administration method combining cashew gum polysaccharide with either benzocaine or chlorhexidine.

This review included four case–control studies conducted between 2005 and 2021 (33–36) (Table 1).

**Table 1:** Study characteristics, clinical periodontal parameters and selected inflammatory markers in four included studies.

Title of the study	Author (Year)	Country	Fiber type	Intervention	Type and number of animals	Induced periodontitis model
Soluble *β*-1,3/1,6-glucan from yeast inhibits experimental periodontal disease in Wistar rats	Breivik et al. (33)	Norway	Soluble *β*-1,3/1,6-glucan (*Saccharomyces cerevisiae*)	10 mg/kg, *ad libitum* in drinking water for 7 weeks	Male wistar rats (*n* = 30)	3/0 Silk ligature, maxillary left second molar
Effects of polycan, a *β*-glucan, on experimental periodontitis and alveolar bone loss in Sprague–Dawley rats	Kim et al. (34)	Korea	Polycan: 1,3/1,6 β-glucan (*Aureobasidium pullulans*)	21.25 mg/kg, 42.5 mg/kg or 85 mg/kg added to distilled water, administered orally daily for 10 days	Male Sprague–Dawley Rats (n = 42)	3/0 Nylon ligature, maxillary left second molar
Effects of the prebiotic mannan oligosaccharide on the experimental periodontitis in rats.	Levi et al. (35)	Brazil	Mannan Oligosaccharide	1 g/kg diet study, *ad libitum* for 6 weeks	Male Wistar Rats (n = 40)	Cotton ligature, mandibular first molars
Experimental periodontal disease triggers coronary endothelial dysfunction in middle-aged rats: preventive effect of a prebiotic *β*-glucan	Silva et al. (36)	Brazil	β (1.3/1.6)-glucan (Highly purified *Saccharomyces cerevisiae*)	50 mg/kg oral gavage daily for 4 weeks	Wistar Rats (n = 20)	Silk ligature, mandibular first molars
Alveolar bone measurements (intervention *vs* control)	TNF-α	IL-1β	IL-10	IFN-γ	TGF-β Family	Inflammatory Cell Infiltration (PMN)
*↓11% ABL.	↓5%	NM	↑36%	NM	*TGF-1β: ↑23%	NM
21.25 mg/kg: ↑12.05% BV **42.5 mg/kg: ↓27.44% ABL and a ↑48.15% BV. **85 mg/kg:↓61.25% ABL and ↑76.38% BV.	*21.25 mg/kg: ↓15.52% **42.5 mg/kg: ↓35.93% **85 mg/kg: ↓41.98%	21.25 mg/kg: ↓11.39% **42.5 mg/kg: ↓28.99% **85 mg/kg: ↓36.77%	NM	NM	NM	21.25 mg/kg: ↓22.25% **42.5 mg/kg: ↓59.11% **85 mg/kg: ↓89.29%
**↓33% ABL *↑68% ABA ↓19% AL, ↓7% alveolar bone level, ↑10% BV and ↓10% BP.	***↓40%	***↓40%	*↓ relative expression	*↓ relative expression	TGF-β: ↑100%	NM
**↓29% ABL and **↓56% ABAL.	**↓29%	↓20%	↑8%	NM	NM	NM

*Statistically significant *p* < 0.05, **Statistically significant *p* < 0.01, ***Statistically significant *p* < 0.001. NM, not measured; ABA, alveolar bone area; ABL, alveolar bone loss; BV, bone volume; BP, bone porosity; IL-β, interleukin-1β; IL-10, interleukin-10; mg/kg, milligram per kilogram; TGF-β, transforming growth factor-β; TNF-α, tumor necrosis factor-α; PMN, polymorphonuclear leukocyte.

### Study characteristics

All four studies were conducted on rat models. Three studies (33, 35, 36) used Wistar rats and one study used Sprague–Dawley rats (34). Periodontitis was induced in all four studies *via* ligation with nylon (34), cotton (35), or silk (33, 36). Two studies induced periodontitis on maxillary second molars (33, 34) and two studies induced periodontitis on mandibular first molars (35, 36).

Three of the four studies used 1,3/1,6 *β*-glucan (33, 34, 36) as the fiber intervention. Two of the studies used *β*-glucan purified from *Saccharomyces cerevisiae* (33, 36) and one study used *β*-glucan purified from *Aureobasidium pullulans* (34)*. β*-glucan was administered either orally *via* distilled water daily (34), oral gavage daily (36) or *ad libitum* within drinking water (33). One study used mannan oligosaccharide (MOS) *ad libitum* as a dietary supplement for 6 weeks (35).

Of the *β*-glucan studies, (33) provided 10 mg/kg *ad libitum* for 7 weeks (33, 34) investigated three different doses, 21.25 mg/kg, 42.5 mg/kg and 85 mg/kg with a duration of 10 days (34), and (36) administered 50 mg/kg for 4 weeks (36).

### Impact of fiber on periodontal status – primary outcome of four studies

Breivik et al. study measured ABL *via* digital radiographs as the distance between the cementoenamel junction (CEJ) and mesial and distal surfaces of the second molars, showing 11% less ABL (*p* = 0.016) in the EP-*β*-glucan group (0.92 ± 0.10 mm) compared to the EP control group (1.03 ± 0.09 mm) (33).

Kim et al. study recorded total ABL was as a sum of seven distances on buccal tooth surfaces measured from cusp tip to alveolar bone (34). There was significantly less total ABL (*p* < 0.01) in the 42.5 mg/kg and 85 mg/kg Polycan groups compared to the experimental periodontitis (EP) control group (34). However, this was displayed graphically, and numerical values were not reported. The distance from the cusp tip to the alveolar bone was also significantly less in the (*p* < 0.01) 42.5 mg/kg (27.4%) and 85 mg/kg (61.3%) Polycan groups, respectively, compared to the EP group. Furthermore, alveolar bone volume was measured as the alveolar bone area on the regions between the first and second molars on longitudinally trimmed samples using a digital image analyzer. Bone volume was significantly higher (*p* < 0.01) in the 42.5 mg/kg (56.11 ± 11.75%) and 85.0 mg/kg (66.80 ± 6.68%) Polycan groups compared to the EP group (37.87 ± 6.46%) (34).

In the (35) study, interproximal ABL was measured as the sum of the distance between the CEJ and the alveolar bone crest at the distal of the first molar, mesial of the second molar and the center of the interproximal area between the first and second molar on the lingual surfaces of defleshed jaws (35). The EP-MOS group (0.89 ± 0.11 mm) showed significantly lesser ABL (*p* < 0.001) compared to the EP group (1.33 ± 0.26 mm). A 33% decrease in ABL (*p* < 0.01) and a 68% increase in Alveolar Bone Area (ABA) (*p* < 0.05) was noted. This study also determined bone volume, bone porosity, trabecular number, trabecular separation, and mean bone mineral density on non-demineralized specimens using cone-beam microcomputed tomography. ABL as a mean of four different sites (buccal, lingual, interproximal and furcation) was significantly less (*p* = 0.000) in the EP-MOS group (3.74 ± 0.81 mm) compared to the EP group (4.04 ± 0.85 mm). Bone porosity measured as a percentage of porosities present in bone tissue was significantly lower (*p* = 0.003) in the EP-MOS group (45.08 ± 15.83%) compared to the EP group (50.43 ± 14.35%). Conversely, mean bone mineral density was significantly higher (*p* = 0.020) in the EP-MOS group (1.45 ± 0.09%) compared to the EP group (1.42 ± 0.05%). Histomorphometric analysis was used to determine attachment loss and area of no bone in the central portion of the bifurcation area and analyzed with light microscopy. Area of no bone was significantly less (*p* < 0.001) in the EP-MOS (0.21 ± 0.03 mm^3^) compared to the EP group (0.65 ± 0.30 mm^3^).

In Silva et al. study, ABL and alveolar bone area loss (ABAL) were measured *via* hematoxylin and eosin staining and slide photography of fixed mandibles (36). ABL was measured as the distance from the CEJ to the alveolar bone crest between the first and second molars and ABAL was measured as the loss of bone area between the interradicular bone crest and furcation. Both parameters were significantly lesser in the EP-*β*-glucan group compared to the EP group (*p* < 0.01) in middle-aged rats.

### Impact of fiber on secondary outcomes

#### Inflammatory biomarkers

##### TNF-α

TNF-α was measured in all four studies (Table 1). Three studies measured TNF-α *via* enzyme-linked immunosorbent assay (ELISA) (33, 34, 36) and one study used immunohistochemistry (IHC) (35). Two studies used serum (33, 36), one used buccal gingiva from the area surrounding the ligature placement (34), and one used immunolabeling of the alveolar bone in the furcation region (35) for analysis. TNF-α was 15.52% (*p* < 0.05), 35.93% (*p* < 0.01) and 41.98% (*p* < 0.01) less in the 21.25 mg/kg, 42.50 mg/kg and 85.00 mg/kg EP-Polycan groups, respectively, compared to EP control group (34). The EP-MOS group displayed lesser immunolabelling TNF-α pattern compared to EP control group (*p* < 0.001) (35). Likewise, (36) reported significantly less TNF-α in the β-glucan group compared to EP group (*p* < 0.01) (showed a 29% decrease in TNF-α concentration) (36).

##### Il-1β

IL-1β was measured *via* ELISA in two studies (34, 36) and IHC in one study (35). Gingival IL-1β was significantly less by 28.99 and 36.77% in the 42.5 mg/kg and 85 mg/kg EP-Polycan groups respectively, compared to EP control group (*p* < 0.01) (34). Likewise, IL-1β labeling in the alveolar bone was significantly less in the EP-MOS group compared to the EP group (35).

##### Il-10

IL-10 was measured *via* ELISA in two studies (33, 36) and qRT-PCR in one study (35). Relative gene expression of IL-10 in gingival tissues was lesser in EP-MOS group compared to EP control group (*p* < 0.05) (35).

##### TGF-1β

Transforming growth factor β (TGF-β) was measured in two studies *via* ELISA (33) and IHC (35). Serum TGF-β was significantly higher in the β-glucan (34.04 ± 5.83 pg./ml) compared to the EP control group (27.78 ± 8.02 pg./ml, *p* = 0.032) (33).

##### IFN-γ

Relative gene expression of gingival interferon-gamma (IFN-γ) measured *via* qRT-PCR was significantly less in the EP-MOS group compared to the EP control group (*p* < 0.05) (35).

#### Other parameters

Other parameters investigated are noted in Supplementary Table S2. Serum corticosterone analyzed *via* ^125^I radioimmunoassay was significantly more (28%, *p* = 0.047) in the β-glucan group (1371.00 ± 308.0 nm) compared to the control group (1067.4 ± 421.6 nm) (33).

Kim et al. (34) study analyzed gingival inducible nitric oxide synthase, myeloperoxidase and malondialdehyde, gingival inflammatory cell infiltrate, collagen fiber occupied regions, osteoclast cells on alveolar bone surface and osteoclast cell occupied regions on alveolar bone surface. All parameters were significantly lower (*p* < 0.01) in the 42.5 mg/kg and 85 mg/kg Polycan groups compared to the EP control group (Supplementary Table S2).

Gut health was assessed by analyzing crypt depth and villous height of the small and large intestines *via* histomorphometry. The EP-MOS group showed normal intestinal structure compared to EP group which displayed reduced villus height (*p* < 0.001) and lower crypt depth (*p* < 0.01) (35).

Silva et al. (36) also analyzed serum hydroperoxide concentration (*via* the ferrous oxidation-xylenol orange assay) and gingival induced nitric oxide synthase, cyclooxygenase 1, cyclooxygenase 2, p47phox, gp91phox, NF-κB p65, p53, p21 and p16 (*via* western blotting). Of these, induced nitric oxide synthase expression was significantly lower in the β-glucan-EP group compared to EP control (*p* < 0.01) (36).

### Quality assessment

All four studies were assessed against the SYRCLE’s framework in determining their respective risks of bias (Table 2). All four studies included appropriate control groups and had clearly stated baseline measurements before intervention initiation. Allocation concealment, sequence generation, random housing, performance blinding, random outcome assessment, and detection bias criteria were not clear in all four studies. Two studies addressed whether any incomplete data occurred and why. Lastly, all four studies addressed any reporting bias’ by stating how selective reporting was examined.

**Table 2:** Risk of bias assessment as adapted from Hooijmans et al. (30).

Characteristics^†^	Breivik et al. (33)	Kim et al. (34)	Levi et al. (35)	Silva et al. (36)
#1: Sequence Generation	?	?	?	?
#2: Baseline Characteristics	+	+	+	+
#3: Allocation Concealment	?	?	?	?
#4: Random Housing	?	?	?	?
#5: Performance Blinding	?	?	?	?
#6: Random Outcome Assessment	?	?	?	?
#7: Detection Blinding	?	?	?	?
#8: Attrition Bias	–	+	+	–
#9: Selective Outcome Reporting	+	+	+	+

^†^Broad characteristics include Selection Bias (#1, #2, & #3), Performance Bias (#4 & #5), Detection Bias (#6 & #7), Attrition Bias (#8) and Reporting Bias (#9) (30). +, yes; −, no; ?, unclear.

The CAMARADES checklist was also utilized to assess reporting quality for all four included studies (Table 3). Four studies were published in a peer-reviewed journals (Journal of Periodontal Research, Journal of Clinical Periodontology, and Journal of Gerontology). All four studies also addressed a controlled temperature for the housing of all animals. Three studies stated that the treatment/control was randomized. Allocation concealment and blinded assessment or outcome criteria were not clear in all four studies. The use of anesthetic with marked intrinsic properties were avoided all four studies. All four studies avoided the use of animals with hypertension or diabetes as per the inclusion criteria. Only one study included a power calculation in evaluating the sample size in the article. All four studies included a statement of compliance with their relative regulatory board and two studies stated any possible conflict of interest.

**Table 3:** Reporting quality checklist as assessed by CAMARADES, adapted from Auboire et al. (31) and Macleod et al. (32).

Quality check list criteria	Breivik et al. (33)	Levi et al. (35)	Silva et al. (36)	Kim et al. (34)
#1 Publication in a peer-reviewed journal	+	+	+	+
#2 Statement of control of temperature	+	+	+	+
#3 Randomization of treatment or control	+	?	+	+
#4 Allocation concealment	?	?	?	?
#5 Blinded assessment of outcome	?	?	?	?
#6 Avoidance of anesthetics with marked intrinsic properties	+	+	+	+
#7 Use of animals with hypertension or diabetes	–	–	–	–
#8 Sample size calculation	?	?	+	?
#9 Statement of compliance with regulatory requirements	+	+	+	+
#10 Statement regarding possible conflict of interest	?	?	+	+
Total	5	4	7	6

+, yes; −, no; ?, unclear.

According to the reporting quality checklist as assessed by CAMARADES Breivik et al. scored 5 out of 10, Kim et al. scored 4 out of 10, Levi et al. scored 7 out of 10 and Silva et al. scored 6 out of 10.

## Discussion

This systematic review investigated for the first time how dietary fiber affects periodontitis in animal models. The four included studies identified that dietary fiber supplementation with *β*-glucan or MOS ameliorates alveolar bone loss associated with periodontitis in rats (33–36). These studies also investigated the effect of dietary fiber on local and systemic inflammatory mediators and one study which had various amounts of fiber alluded to a dose-dependent relationship. All four studies did not examine the role of microbiota or their metabolites such as SCFA on this immune-modulatory behavior of dietary fiber. However, gut health was assessed by (35) where the use of MOS as an intervention preserved crypt depth and villous height in the gut indicating a healthier gut morphology (35).

Studies have shown that fiber reduces the risk of non-communicable diseases as diets rich in fiber have antioxidants and less saturated fat (37). (36) examined the secondary effect of periodontitis-induced endothelial dysfunction and found that prebiotic *β*-glucan in addition to the reduction of periodontal parameters ABL and ABAL, also delayed coronary artery endothelial dysfunction by preventing oxidative stress, reducing circulating pro-inflammatory cytokines and inducible nitric oxide synthase suggesting a beneficiary impact on the coronary vascular bed (36). This suggests that although the prebiotic *β*-glucan has benefits for periodontal disease, it may also contribute to reducing other systemic inflammatory conditions that are exacerbated by the presence of periodontitis. Periodontitis is bidirectionally associated with cardiometabolic disorders and epidemiologically linked with a range of other systemic disorders including rheumatoid arthritis, liver diseases and chronic kidney disease (38). Consequently a recent systematic review found that management of periodontitis results in systemic health improvements in cardiometabolic risk, occurrence of preterm deliveries and a decrease in systemic inflammatory profile (39).

The ingestion of dietary fiber has been suggested to influence systemic inflammatory markers (40). Moreover, there is less accumulation of pro-inflammatory cytokines IL-1β and TNF-α in the local periodontal tissue following an intake of dietary fiber by-products such as β-glucans and MOS (34–36). The release of local inflammatory markers has an effect of distant systemic sites and this is also identified in the periodontal disease (41, 42). The study identifies a trend of less systemic pro-inflammatory cytokines with the addition of certain prebiotics orally in rats (34–36). Although the data was not significant, a trend is noticed for a dose-dependent relationship for *β*-glucans and the anti-inflammatory marker IL-10 (33, 36). Although this anti-inflammatory effect is suggested with β-glucan supplementation, the opposite is seen when MOS is administered producing significantly lower expression of IL-10 relative to the experimental periodontitis model, questioning the key differences between certain sub-cellular structures of prebiotics and their use as an anti-inflammatory cytokine modulator (35).

Mannan oligosaccharide is primarily found in yeast cell walls and hence, is not part of a mainstream dietary fiber source for humans. However, it is still part of the dietary fiber food group as it is a prebiotic and is used in the animal industry as a food supplement to improve economic performance and reduce pathogenic contamination as it is resistant to digestive enzymes in the gut (43). A recent study in piglets found that MOS can improve the gut morphology by increasing the duodenal villi height and pro-inflammatory markers like TNF-α (44). Of note is the reduction in Toll Like Receptor 4 activation where MOS competes with LPS as a substrate. Similar results were found by Levi et al. (35) within this systematic review, improving gut morphology and reducing systemic pro-inflammatory factors from the intake of dietary fibers (35).

Anaerobic bacteria inhabiting the colon ferment fiber and produce short-chain fatty acids. Research has shown resistant starch is the most potent SCFA producer from dietary fiber (45, 46) and these SCFAs are a vital energy source for the gut epithelial cells strengthening the gut barrier. Apart from the immuno-modulatory effect of dietary fiber, the gut and digestive benefits that have been explored in previous studies have also been backed up by the study conducted by Levi et al. (35). This study involving MOS showed a significant increase in both villous height and crypt depth, leading to greater functioning of the digestive health in rats (35). There is a relationship between a dose-dependent increase in certain dietary fiber sub-components and the reduction in periodontal disease, however the mechanism involved is yet to be determined (34) since none of the included papers examined the gut microbiome and determined its changes over time or measured SCFAs.

Prebiotics modulate periodontitis in laboratory animals. Although this systematic review shows an impact in favor of the fiber treated experimental group, these results should be interpreted with care due to the small number of articles included. In human, bleeding on probing, CAL, probing depth, plaque index and gingival index are reflective clinical signs of periodontal inflammation. However, this systematic review looks further than clinical values and shows a decreasing trend of the periodontal markers ABL, ABAL, bone volume, IL-1β and TNF-α with an increasing dose of the *β*-glucan fiber producing a clear linear dose-dependent relationship between fiber intake and the above mentioned periodontal parameters (33, 34, 36). Furthermore, the attachment loss measurement by (35), although not significant, explored the loss of interdental papilla, apical migration of the junctional epithelium, disorganization and disruption of transeptal collagen fibers, the presence of moderate to severe mononuclear inflammation at both the subepithelial connective tissue and at the margins of the periodontal ligament as well as any loss of interdental cementum and reabsorption of the alveolar bone, going above the level of measurement in the other three studies (35).

These periodontal parameters are limited in scope. Recent research has involved the role of micro-RNA (miRNA), messenger RNA (mRNA) and transglutaminases in periodontal parameters which can ultimately aid in diagnosing periodontitis within the early stages (16, 17, 47). In particular, the link between the gut microbiome and periodontal severity can be measured *via* the expression of mRNA and protein expression of Claudin-1 and Claudin-2 (17). Hence future direction in pre-clinical trials warrants for the inclusion of miRNA, mRNA and transglutaminases in further diagnosing the severity of periodontitis in response to a dose dependent change in dietary fiber intervention. The inclusion of a meta transcriptome analysis can provide more details including the type of microbes inhabiting the gut and their functions, including the type of RNA expression. PDL collagen morphology and the levels of transglutaminase are a powerful tool that can be analyzed to determine the makeup of the periodontal ligament in various stages of periodontitis, producing either a thin or thick morphology (47). This parameter has not been assessed yet in response to an intake of dietary fiber, and although once there is a clinical loss of alveolar bone and a clear loss of periodontal ligament structure in severe periodontitis, the type of periodontal ligament, whether thick or thin can provide useful information for the treatment and management of patients in the diagnosis and treatment of periodontitis utilizing an individual-based approach to patient centered care.

The average recommended daily intake of dietary fiber is 25 g/day for adult women and 30 g/day for adult men (21). However, studies have shown that the amount of dietary fiber intake still remains low. Therefore, the recommended dose of prebiotics by these studies of *β*-glucans and MOS is too high to be implemented as part of a human diet. This alludes toward the need of a broader dietary fiber study to be completed incorporating many different sub-cellular components that are readily found in the human dietary fiber diet.

Although this systematic review evaluates the use of pre-biotics as an alternative therapeutic modality to managing periodontitis, there are confounding limitations. For example, out of the 3,950 studies screened, the four studies included were all rat models. Using rats is great for predictability in inducing the infectious disease model correctly *via* ligation as well as its similarities in molar morphology to humans, however rodents are naturally resistant to periodontitis and have a different microbiota to humans (48). It is more appropriate to draw parallels between an accurate representation of periodontal disease progression in a naturally occurring model of periodontitis utilizing the putative periodontal pathogens commonly found in the human model of periodontitis progression compared to a cotton/silk/nylon retentive thread ligation. Similarly, more studies using different animal models are warranted, to know whether this effect is similar in other animal models before moving into clinical trials.

The pool of prebiotics explored in this systematic review is underwhelming, resulting in four studies that fit the criteria as described. Hence it is not appropriate to extrapolate the findings into the broader category of dietary fiber, and further pre-clinical studies are mandated before data can be interpreted and moved into large-scale clinical trials. Dietary fiber includes all parts of plant-derived food that cannot be broken down enzymatically in the gut. By analyzing only two sub-cellular components of dietary fiber, *β*-glucans and MOS, an accurate depiction of dietary fiber cannot be established within the current literature.

The reduction in periodontitis severity with the intervention of *β*-glucans and MOS shows promise in the use of prebiotics as a therapeutic agent. These sub-cellular components make up the cell wall in indigestible plant matter, a part of dietary fiber and useful knowledge for the future of dietary studies in managing chronic pro-inflammatory conditions.

## Conclusion

The results of all four studies are promising in the use of subcellular prebiotics. *β*-glucans derived from the cell wall of *Saccharomyces cerevisiae* and *Aureobasidium pullulans*, and mannan oligosaccharide, successfully reduced the amount of alveolar bone loss in a dose-dependent manner. Pro-inflammatory cytokine levels reduced locally and systemically in a periodontitis model, suggesting the use of these prebiotics as a therapeutic alternative to managing the severity of periodontitis.

However, these results do not uncover the link between the production of short-chain fatty acids from dietary fiber and its immuno-modulatory effect on the chronic inflammatory disease periodontitis. Future directions to uncover this link between dietary fiber and its systemic therapeutic effect on oral periodontal disease, requires preclinical studies with a higher diversity of dietary fiber within the normal range of dietary fiber intake, as well as a more robust set of biomarkers that can help diagnose and treat oral and periodontal diseases early in the disease progression.

## Data availability statement

The original contributions presented in the study are included in the article/Supplementary material, further inquiries can be directed to the corresponding author.

## Author contributions

RT, JE, and TJ contributed to the conception and design of the manuscript. RT and TJ filtered articles for this systematic review and wrote the manuscript. RT, NM, AC, HS, JE, and TJ critically analyzed the content and edited the manuscript. All authors reviewed and approved the final version of the manuscript, read and agreed to the published version of the manuscript.

## Conflict of interest

The authors declare that the research was conducted in the absence of any commercial or financial relationships that could be construed as a potential conflict of interest.

## Publisher’s note

All claims expressed in this article are solely those of the authors and do not necessarily represent those of their affiliated organizations, or those of the publisher, the editors and the reviewers. Any product that may be evaluated in this article, or claim that may be made by its manufacturer, is not guaranteed or endorsed by the publisher.

## Abbreviations

ABA: alveolar bone area
ABAL: alveolar bone area loss
ABL: alveolar bone loss
CAL: clinical attachment loss
CEJ: cementoenamel junction
ELISA: enzyme-linked immunosorbent assay
EP: experimental periodontitis
IFN-γ: interferon gamma
IHC: immunohistochemistry
IL: interleukin
LPS: lipopolysaccharide
MOS: mannan oligosaccharides
SCFA: short-chain fatty acids
TGF-β: transforming growth factor beta
T-Reg: t-regulatory cell
TNF-α: tumor necrosis factor – alpha
