# Supplementary material for: Role of prebiotic dietary fiber in periodontal disease: A systematic review of animal studies
Source: Front Nutr. 2023 Mar 14;10:1130153. doi: 10.3389/fnut.2023.1130153 (PMC10043215; doi:10.3389/fnut.2023.1130153)
Supplement: Supplementary file 1 [file Table_1.docx]

**SUPPLEMENTARY MATERIAL**

**Supplementary table 1:** Electronic search strategy

| Database type | Search query |
| --- | --- |
| Medline | 75. Periodontitis/ 76. Periodontitis.mp. 77. periodont* health.mp. 78. Periodontal Diseases/ 79. periodont* disease*.mp. 80. Dental disease*.mp. 81. Gum disease*.mp. 82. Gingival Diseases/ 83. Gingi* disease*.mp. 84. Tooth disease*.mp. 85. Tooth health.mp. 86. Bone loss*.mp. 87. Chronic Periodontitis/ 88. Chronic Periodontitis.mp. 89. Chronic Periodontitis/90. Periodontalinflammat*.mp. 91. Gingivitis/ 92. Gingivitis.mp. 93. Gingival Inflammat*.mp. 94. Aggressive Periodontitis/ 95. Aggressive periodontitis.mp. 96. Pocket depth.mp. 97. Periodontal Attachment Loss/ 98. Periodontal Attachment Loss*.mp. 99. Clinical attachment loss*.mp. 100. "Bleeding on probing".mp. 101. Clinical probing depth.mp. 102. Gingival bleed*.mp. 103. Periodontal Ligament/ 104. Periodontal ligament*.mp. 105. Gingival Crevicular Fluid/ 106. Gingival crevicular fluid*.mp. 107. Porphyromonas gingivalis/ 108. Porphyromonas gingivalis.mp. 109. exp Periodontal Atrophy/ 110. (Periodontal Atrophy or Gingival Recession or Alveolar Bone Loss).mp. 164. dental plaque.mp. or Dental Plaque/ 165. Gingiva/ 166. gingiv*.mp.169. periodontal pocket.mp. or Periodontal Pocket/ 170. periodontal abscess.mp. or Periodontal Abscess/  111. Dietary Fiber/ 112. (Fibre or Fiber or Fibrous).mp. 113. Prebiotics/ 114. prebiotic*.mp. 115. Inulin/ 116. Inulin.mp. 117. Resistant Starch/ 118. resistant starch.mp. 119. Pectins/ 120. pectin*.mp. 121. Cellulose/ 122. cellulose*.mp. 123. Lignin/ 124. Lignin.mp. 125. Gum.mp. 126. Plant Mucilage/ 127. Plant Mucilage.mp. 128. Plant Mucilage.mp. 129. Roughage.mp. 130. Hemicellulose*.mp. 131. Psyllium/ 132. Psyllium.mp. 133. beta-Glucans/ 134. beta-Glucans.mp. 135. Whole Grains/ 136. Whole grain*.mp. 137. Bran.mp. 138. Hemicellulose.mp. 139. Crude fibre.mp. 140. soluble fibre.mp. 141. fermentable fibre.mp. 142. insoluble fibre.mp. 143. Dextrins/ 144. Dextrin*.mp 145. Oligosaccharides/ 146. oligosaccharide*.mp. 147. wheat bran.mp. 148. Polysaccharides/ 149. polysaccharide*.mp. 150. non-starch polysaccharide*.mp. 151 Chitin/ 152. Arabinoxylan.mp. 153. Fructans/ 154.fructan*.mp. 155. Polyuronide.mp. 156. Raffinose/ 157. Raffinose.mp. 158. Polydextrose.mp. 159. xanthan gum.mp. 160. guar gum.mp. 161. Plant Gums/ 162. Plant gum*.mp. 163. periodont* pathogen*.mp. 171. methylcellulose.mp. or Methylcellulose/ 172. Arabinogalactan.mp. 173. Galactomannan.mp. 179. Chitin.mp.  167. Animals/ 168. animal*.mp.  174. 75 or 76 or 77 or 78 or 79 or 80 or 81 or 82 or 83 or 84 or 85 or 86 or 87 or 88 or 89 or 90 or 91 or 92 or 93 or 94 or 95 or 96 or 97 or 98 or 99 or 100 or 101 or 102 or 103 or 104 or 105 or 106 or 107 or 108 or 109 or 110 or 163 or 164 or 165 or 166 or 169 or 170  175. 111 or 112 or 113 or 114 or 115 or 116 or 117 or 118 or 119 or 120 or 121 or 122 or 123 or 124 or 125 or 126 or 127 or 128 or 129 or 130 or 131 or 132 or 133 or 134 or 135 or 136 or 137 or 138 or 139 or 140 or 141 or 142 or 143 or 144 or 145 or 146 or 147 or 148 or 149 or 150 or 151 or 152 or 153 or 154 or 155 or 156 or 157 or 158 or 159 or 160 or 161 or 162 or 171 or 172 or 173  177. 167 or 168  180. 175 or 179  181. 174 and 177 and 180 |
| Embase | 1. Periodontitis.mp. or periodontitis/ 2. periodont* health.mp. 3.  periodontal disease/ or periodont* disease.mp. 4. Dental disease*.mp. 5. Gum disease*.mp. 6. gingiva disease/ or Gingi* disease*.mp. 7. Tooth disease.mp. or tooth disease/ 8. Tooth health.mp. 9. Bone loss*.mp. 10 Chronic Periodontitis.mp. or chronic periodontitis/ 11. Periodontal inflammat*.mp. 12. Gingivitis.mp. or gingivitis/ 13. Gingival Inflammat*.mp. 14. Aggressive periodontitis.mp. or aggressive periodontitis/ 15. Pocket depth.mp. 16. Periodontal Attachment Loss*.mp. 17. Clinical attachment loss*.mp. 18. "Bleeding on probing".mp. 19. Clinical probing depth.mp. 20. gingiva bleeding/ or Gingival bleed*.mp. 21. periodontal ligament/ or Periodontal ligament*.mp. 22. Gingival crevicular fluid*.mp. 23. Porphyromonas gingivalis.mp. or Porphyromonas gingivalis/ 24. Periodontal Atrophy.mp. 25. Gingival Recession.mp. 26. Alveolar Bone Loss.mp. or alveolar bone loss/ 62. periodont* pathogen*.mp. 63. dental plaque.mp. or tooth plaque/ 64. gingiva disease/ or gingiva/ or gingivitis/ or gingiv*.mp. 71. periodontal pocket.mp. or periodontal pocket/ 72. periodontal abscess.mp. or periodontal abscess/  27. Fibre.mp. 28. Fiber.mp. or dietary fiber/ or fiber/ 29. Fibrous.mp. 30. prebiotic*.mp. 31. inulin/ or Inulin.mp. 32. resistant starch.mp. 33. pectin/ or pectin*.mp. 34. cellulose*.mp. or cellulose/ 35. lignin/ or lignin.mp. 36. Gum.mp. 37. Plant Mucilage.mp. or mucilage/ 38. roughage.mp. or roughage/ 39. hemicellulose/ or Hemicellulose*.mp. 40 psyllium.mp. or ispagula/ 41. beta-Glucans.mp. or beta glucan/ 42. Whole grain*.mp. or whole grain/ 43. bran/ or Bran.mp. 44 hemicellulose/ or Hemicellulose.mp. 45. Crude fibre.mp. 46. soluble fibre.mp. 47. fermentable fibre.mp. 48. insoluble fibre.mp. 49. dextrin/ or Dextrin*.mp. 50. oligosaccharide/ or oligosaccharide*.mp. 51 wheat bran.mp. or wheat bran/ 52. polysaccharide/ or polysaccharide*.mp. 53. non-starch polysaccharide*.mp. 54. arabinoxylan.mp. or arabinoxylan/ 55. fructan/ or fructan*.mp. 56. Polyuronide.mp. 57. raffinose.mp. or raffinose/ 58. Polydextrose.mp. 59. xanthan gum.mp. 60. guar gum.mp. or guar gum/ 61. plant gum/ or Plant gum*.mp. 73. methylcellulose.mp. or methylcellulose/ 74. arabinogalactan.mp. or arabinogalactan/ 75. galactomannan.mp. or galactomannan/  Animal: 6198265  69. animal/ or animal*.mp.  Perio: 223,788  76. 1 or 2 or 3 or 4 or 5 or 6 or 7 or 8 or 9 or 10 or 11 or 12 or 13 or 14 or 15 or 16 or 17 or 18 or 19 or 20 or 21 or 22 or 23 or 24 or 25 or 26 or 62 or 63 or 64 or 71 or 72  Fibre: 872,460  77. 27 or 28 or 29 or 30 or 31 or 32 or 33 or 34 or 35 or 36 or 37 or 38 or 39 or 40 or 41 or 42 or 43 or 44 or 45 or 46 or 47 or 48 or 49 or 50 or 51 or 52 or 53 or 54 or 55 or 56 or 57 or 58 or 59 or 60 or 61 or 73 or 74 or 75  Combined with AND: 1,868  78. 69 and 76 and 77 |
| Cinahl | S1 (MH "Periodontitis+") OR "periodontitis" S2 "periodont* health" S3 (MH "Periodontal Diseases+") OR "periodont* disease*" S4 "Dental disease*" S5 (MH "Gingival Diseases+") OR "Gum disease*" S6 "Gingi* disease*" S7 (MH "Tooth Diseases+") OR "Tooth disease*" S8 "Tooth health" S9 "Bone loss*" S10 (MH "Chronic Periodontitis") OR "Chronic Periodontitis" S11 "Periodontal inflammat*" S12 (MH "Gingivitis+") OR "Gingivitis" S13 "Gingival Inflammat*" S14 (MH "Aggressive Periodontitis") OR "Aggressive periodontitis" S15 "Pocket depth" S16 (MH "Periodontal Attachment Loss") OR "Periodontal Attachment Loss*" S17 "Clinical attachment loss*" S18 "Bleeding on probing" S19 "Clinical probing depth" S20 "Gingival bleed*" S21 (MH "Periodontal Ligament") OR "Periodontal ligament*" S22 "Gingival crevicular fluid*" S23 "porphyromonas gingivalis" S24 (MH "Periodontal Atrophy+") OR "Periodontal Atrophy" S25 (MH "Gingival Recession") S26 (MH "Alveolar Bone Loss") S27 "periodont* pathogen*" S28 "dental plaque" S29 (MH "Gingiva+") OR "gingiv*" S68 "Gingival Recession" S69 "Alveolar bone loss" S70 (MH "Dental Plaque") S74 (MH "Periodontal Pocket") OR "periodontal pocket" S75 (MH "Periodontal Abscess") OR "periodontal abscess"  S30 "Fibre" S31 "Fiber" S32 "Fibrous" S33 (MH "Prebiotics") S34 "Inulin" S35 (MH "Resistant Starch") OR "resistant starch" S36 "pectin*" S37 (MH "Cellulose") OR "cellulose*" S38 "lignin" S39 "gum" S40 "Plant Mucilage" S41 "roughage" S42 "Hemicellulose*" S43 (MH "Psyllium") OR "psyllium" S44 (MH "Beta-Glucans") OR "beta-Glucans" S45 "Whole grain*" S46 "Bran" S47 "Crude fibre" S48 "soluble fibre" S49 "fermentable fibre" S50 "insoluble fibre" S51 "Dextrin*" S52 (MH "Oligosaccharides+") S53 "oligosaccharide*" S54 "wheat bran" S55 (MH "Polysaccharides+") OR "polysaccharide*" S56 "non-starch polysaccharide*" S57 "arabinoxylan" S58 "fructan*" S59 "polyuronide" S60 "polyuronide" S61 "raffinose" S62 "polydextrose" S63 "xanthan gum" S64 "guar gum" S65 (MH "Plant Gums+") OR "Plant gum*" S66 "Methylcellulose" S67 "Arabinogalactan" S72 "prebiotic*" S73 "galactomannan"  S71 "animal*" OR (MH "Animals+"): 258,728  Perio: 68,482  S76 S1 OR S2 OR S3 OR S4 OR S5 OR S6 OR S7 OR S8 OR S9 OR S10 OR S11 OR S12 OR S13 OR S14 OR S15 OR S16 OR S17 OR S18 OR S19 OR S20 OR S21 OR S22 OR S23 OR S24 OR S25 OR S26 OR S27 OR S28 OR S29 OR S68 OR S69 OR S70 OR S74 OR S75  Fibre: 69,207  S77 S30 OR S31 OR S32 OR S33 OR S34 OR S35 OR S36 OR S37 OR S38 OR S39 OR S40 OR S41 OR S42 OR S43 OR S44 OR S45 OR S46 OR S47 OR S48 OR S49 OR S50 OR S51 OR S52 OR S53 OR S54 OR S55 OR S56 OR S57 OR S58 OR S59 OR S60 OR S61 OR S62 OR S63 OR S64 OR S65 OR S66 OR S67 OR S72 OR S73  S78 S71 AND S76 AND S77 |
| Web of Science | Perio:132,329  #4 (ALL=(“periodontal pocket”)) OR ALL=(“periodontal abscess”)) OR #1  #1 ((((((((((((((((((((((((((((((ALL=(Periodontitis)) OR ALL=("periodont* health")) OR ALL=("periodont* disease*")) OR ALL=(“Dental disease*”)) OR ALL=("Gum disease*")) OR ALL=("Gingi* disease*")) OR ALL=("Tooth disease*")) OR ALL=("Tooth health")) OR ALL=("Bone loss*")) OR ALL=("Chronic Periodontitis")) OR ALL=("Periodontal inflammat*")) OR ALL=(Gingivitis)) OR ALL=("Gingival Inflammat*")) OR ALL=("Aggressive periodontitis")) OR ALL=("Pocket depth")) OR ALL=("Periodontal Attachment Loss*")) OR ALL=("Clinical attachment loss*")) OR ALL=("Bleeding on probing")) OR ALL=("Clinical probing depth")) OR ALL=("Gingival bleed*")) OR ALL=("Periodontal ligament*")) OR ALL=("Gingival crevicular fluid*")) OR ALL=("Porphyromonas gingivalis")) OR ALL=("Periodontal Atrophy")) OR ALL=("Gingival Recession")) OR ALL=("Alveolar Bone Loss")) OR ALL=("periodont* pathogen*")) OR ALL=("dental plaque")) OR ALL=(gingiv*)))  Fibre: 1,523,178  #5 (((ALL=(methylcellulose)) OR ALL=(arabinogalactan)) OR ALL=(galactomannan)) OR #2  #2 ((((((((((((((((((((((((((((((((((ALL=(Fibre)) OR ALL=(Fiber)) OR ALL=(Fibrous)) OR ALL=(prebiotic*)) OR ALL=(Inulin)) OR ALL=("resistant starch")) OR ALL=(pectin*)) OR ALL=(cellulose*)) OR ALL=(lignin)) OR ALL=(gum)) OR ALL=("Plant Mucilage")) OR ALL=(roughage)) OR ALL=(Hemicellulose*)) OR ALL=(psyllium)) OR ALL=(beta-Glucans)) OR ALL=("Whole grain*")) OR ALL=(Bran)) OR ALL=(Hemicellulose)) OR ALL=("Crude fibre")) OR ALL=("soluble fibre")) OR ALL=("fermentable fibre")) OR ALL=("insoluble fibre")) OR ALL=(Dextrin*)) OR ALL=(oligosaccharide*)) OR ALL=("wheat bran")) OR ALL=(polysaccharide*)) OR ALL=("non-starch polysaccharide*")) OR ALL=(arabinoxylan)) OR ALL=(fructan*)) OR ALL=(polyuronide)) OR ALL=(raffinose)) OR ALL=(polydextrose)) OR ALL=("xanthan gum")) OR ALL=("guar gum")) OR ALL=("Plant gum*")  #3 ALL=(animal*): [1,605,287](https://www-webofscience-com.ezproxy.library.sydney.edu.au/wos/woscc/summary/cf6c1ccb-890c-4b85-879c-eab2fcf34949-07a49f52/relevance/1)  Final Number combined with ALL:  ((#4) AND #5) AND #3: 553  <https://www.webofscience.com/wos/woscc/summary/b9c63503-cfc2-43ff-946c-408070e4f591-08bbe65c/relevance/1> |
| Scopus | ( TITLE-ABS-KEY ( periodontitis )  OR  TITLE-ABS-KEY ( "periodont* health" )  OR  TITLE-ABS-KEY ( "periodont* disease*" )  OR  TITLE-ABS-KEY ( "Dental disease*" )  OR  TITLE-ABS-KEY ( "gum disease" )  OR  TITLE-ABS-KEY ( "gingi* disease*" )  OR  TITLE-ABS-KEY ( "tooth disease*" )  OR  TITLE-ABS-KEY ( "tooth health" )  OR  TITLE-ABS-KEY ( "bone loss*" )  OR  TITLE-ABS-KEY ( "chronic periodontitis" )  OR  TITLE-ABS-KEY ( "periodontal inflammat*" )  OR  TITLE-ABS-KEY ( gingivitis )  OR  TITLE-ABS-KEY ( "gingival inflammat*" )  OR  TITLE-ABS-KEY ( "aggressive periodontitis" )  OR  TITLE-ABS-KEY ( "pocket depth" )  OR  TITLE-ABS-KEY ( "periodontal attachment loss*" )  OR  TITLE-ABS-KEY ( "clinical attachment loss*" )  OR  TITLE-ABS-KEY ( "bleeding on probing" )  OR  TITLE-ABS-KEY ( "clinical probing depth" )  OR  TITLE-ABS-KEY ( "gingival bleed*" )  OR  TITLE-ABS-KEY ( "periodontal ligament*" )  OR  TITLE-ABS-KEY ( "gingival crevicular fluid*" )  OR  TITLE-ABS-KEY ( "porphyromonas gingivalis" )  OR  TITLE-ABS-KEY ( "periodontal atrophy" )  OR  TITLE-ABS-KEY ( "gingival recession" )  OR  TITLE-ABS-KEY ( "alveolar bone loss" )  OR  TITLE-ABS-KEY ( "periodont* pathogen*" )  OR  TITLE-ABS-KEY ( "dental plaque" )  OR  TITLE-ABS-KEY ( gingiv* )  OR  TITLE-ABS-KEY ( "periodontal abscess" )  OR  TITLE-ABS-KEY ( "periodontal pocket" ) )  AND  ( TITLE-ABS-KEY ( fibre )  OR  TITLE-ABS-KEY ( fiber )  OR  TITLE-ABS-KEY ( fibrous )  OR  TITLE-ABS-KEY ( prebiotic* )  OR  TITLE-ABS-KEY ( inulin )  OR  TITLE-ABS-KEY ( "resistant starch" )  OR  TITLE-ABS-KEY ( pectin* )  OR  TITLE-ABS-KEY ( cellulose* )  OR  TITLE-ABS-KEY ( lignin )  OR  TITLE-ABS-KEY ( gum )  OR  TITLE-ABS-KEY ( "plant mucilage" )  OR  TITLE-ABS-KEY ( roughage )  OR  TITLE-ABS-KEY ( hemicellulose* )  OR  TITLE-ABS-KEY ( psyllium )  OR  TITLE-ABS-KEY ( "beta-glucans" )  OR  TITLE-ABS-KEY ( "whole grain*" )  OR  TITLE-ABS-KEY ( bran )  OR  TITLE-ABS-KEY ( "crude fibre" )  OR  TITLE-ABS-KEY ( "soluble fibre" )  OR  TITLE-ABS-KEY ( "fermentable fibre" )  OR  TITLE-ABS-KEY ( "insoluble fibre" )  OR  TITLE-ABS-KEY ( dextrin* )  OR  TITLE-ABS-KEY ( oligosaccharide* )  OR  TITLE-ABS-KEY ( "wheat bran" )  OR  TITLE-ABS-KEY ( polysaccharide* )  OR  TITLE-ABS-KEY ( "non-starch polysaccharide*" )  OR  TITLE-ABS-KEY ( arabinoxylan )  OR  TITLE-ABS-KEY ( fructan* )  OR  TITLE-ABS-KEY ( polyuronide )  OR  TITLE-ABS-KEY ( raffinose )  OR  TITLE-ABS-KEY ( polydextrose )  OR  TITLE-ABS-KEY ( "xantham gum" )  OR  TITLE-ABS-KEY ( "guar gum" )  OR  TITLE-ABS-KEY ( "plant gum*" )  OR  TITLE-ABS-KEY ( methylcellulose )  OR  TITLE-ABS-KEY ( arabinogalactan )  OR  TITLE-ABS-KEY ( galactomannan ) )  AND  ( TITLE-ABS-KEY ( animal* ) ) |

**Supplementary table 2:** Other parameters investigated in the four explored studies*.*

| **Title of the study** | **Author (Year)** | **iNOS** | **MPO** | **MDA** | **Inflammatory Cell Infiltration (PMN)** | **Collagen Fibre Occupied Region** | **Osteoclast Cells on Alveolar Bone Surface** | **Osteoclast Cell Occupied Regions on Alveolar Bone Surface** | **Villous Height** | **Crypt Depth** | **Corticosterone (nm/l–2 h after i.p. LPS, day of sacrifice)** |
| --- | --- | --- | --- | --- | --- | --- | --- | --- | --- | --- | --- |
| Soluble β-1,3/1,6-glucan from yeast inhibits experimnetal periodontal disease in Wistar rats | Breivik et al (2005) | NM | NM | NM | NM | NM | NM | NM | NM | NM | *↑28% |
| Effects of Polycan, a β-glucan, on experimental periodontitis and alveolar bone loss in Sprague-Dawley rats | Kim et al (2012) | 21.25mg/kg: ↓13.00% **42.5mg/kg: ↓28.18%  **85mg/kg: ↓59.36% | 21.25mg/kg: ↓13.33% **42.5mg/kg: ↓34.67%  **85mg/kg: ↓51.81% | 21.25mg/kg: ↓11.63% **42.5mg/kg: ↓28.91%  **85mg/kg: ↓40.20% | 21.25mg/kg: ↓22.25% **42.5mg/kg: ↓59.11% **85mg/kg: ↓89.29% | 21.25mg/kg: ↑14.63% **42.5mg/kg: ↑71.67% **85mg/kg: ↑104.64% | 21.25mg/kg: ↓14.29% *42.5mg/kg: ↓33.33% **85mg/kg: ↓48.02% | 21.25mg/kg: ↓14.00% **42.5mg/kg: ↓59.86% **85mg/kg: ↓69.99% | NM | NM | NM |
| Effects of the prebiotic mannan oligosaccharide on the experimental periodontitis in rats. | Levi et al (2018) | NM | NM | NM | NM | NM | NM | NM | ***↑25% | **↑50% | NM |
| Experimental Periodontal Disease Triggers Coronary Endothelial Dysfunction in Middle-Aged Rats: Preventive Effect of a Prebiotic β-Glucan | Silva et al (2021) | **↓54% | NM | NM | NM | NM | NM | NM | NM | NM | NM |

**** - Statistically significant p<0.05, ** - Statistically significant p<0.01, *** - Statistically significant p<0.001, NM - Not Measured,*** iNOS – inducible nitric oxide synthase, i. p. - , LPS – lipopolysaccharide, MDA – malondialdehyde, MPO - myeloperoxidase, mg/kg – milligrams per kilogram, nm - , PMN – polymorphonuclear leukocyte. iNOS was measured via liquid scintillation counter of [^3^H]L-citrulline activity. MDA and MPO activity was analysed via spectrophotometric assays. The number of infiltrated inflammatory cells (numbers/mm2 of gingival tissue) and collagen-occupied regions (%/mm2 of gingival tissues) were analysed by a digital image analyser on the gingival areas between the first and second molars via histomorphometry.
